# Development and Validation of an HPLC Method for Analysis of Topotecan in Human Cerebrospinal Fluid and Its Application in Elimination Evaluation of Topotecan after Intraventricular Injection

**DOI:** 10.3390/cancers13184643

**Published:** 2021-09-16

**Authors:** Naoki Yoshikawa, Ai Yamada, Tsubasa Yokota, Yusei Yamada, Mariko Kinoshita, Hiroshi Moritake, Ryuji Ikeda

**Affiliations:** 1 Department of Pharmacy, University of Miyazaki Hospital, 5200 Kihara, Kiyotake-cho, Miyazaki 889-1692, Japan; tsubasa_yokota@med.miyazaki-u.ac.jp (T.Y.); yusei_yamada@med.miyazaki-u.ac.jp (Y.Y.); 2 Division of Pediatrics, Faculty of Medicine, University of Miyazaki, 5200 Kihara, Kiyotake-cho, Miyazaki 889-1692, Japan; ai_yamada@med.miyazaki-u.ac.jp (A.Y.); mariko_kinoshita@med.miyazaki-u.ac.jp (M.K.); hiroshi_moritake@med.miyazaki-u.ac.jp (H.M.)

**Keywords:** cerebrospinal fluid, high-performance liquid chromatography, topotecan, personalized medicine, pharmacokinetics

## Abstract

**Simple Summary:**

Intrathecal administration of anticancer drugs is an effective strategy to treat meningeal leukemia and lymphoma. Topotecan is an anticancer drug, and its anticancer effect is expected via intrathecal administration. In this study, we developed a method to measure the concentration of topotecan in the cerebrospinal fluid (CSF) for clinical monitoring of personalized medicine. By controlling the unique structural feature of topotecan, a pH-dependent lactone ring-opening/closing reversible reaction, we established a simple high-performance liquid chromatography (HPLC) method to measure the total topotecan concentration in the CSF and confirmed that this method can be applied to clinical samples. In the present situation, wherein the concentrations were monitored, the disappearance of topotecan in the CSF was delayed, and signs of adverse effects were confirmed when a high concentration of topotecan was observed.

**Abstract:**

Intrathecal administration of anticancer drugs is an effective dosage strategy, but the elimination of intraventricular drugs is not uniform in all patients. For safety, a system to evaluate local pharmacokinetics in the ventricles after administration is desired. In this study, we developed a simple and reproducible method to measure topotecan concentration in the cerebrospinal fluid (CSF) and confirmed its clinical applicability. High-performance liquid chromatography (HPLC) analysis was performed using a C18 column to measure the total topotecan concentration in the CSF. Clinical CSF samples were obtained from a 1-year old child with poor CSF absorption and stagnation. The patient received topotecan via an intraventricular subcutaneous reservoir. The HPLC method complied with the validation criteria. The lower limit of quantitation of this method was 0.04 µM. Using the developed method, we could determine the difference in topotecan CSF concentrations at 24 and 48 h after administration. The patient’s topotecan elimination rate was extremely low, and signs of adverse effects were observed at high CSF concentration of topotecan. The developed method could detect the delay in topotecan elimination after intrathecal injection. The findings of this study are valuable for the development of personalized treatments for the intrathecal administration of anticancer drugs.

## 1. Introduction

The intrathecal administration of anticancer drugs has the advantage of maintaining high drug concentrations in the cerebrospinal fluid (CSF) and minimizing systemic adverse effects [1]. It is an effective strategy for the first-line treatment and prevention of meningeal leukemia and lymphoma. However, only a limited number of drugs, primarily methotrexate and cytarabine, have been found to be both safe and efficacious when administered via the intrathecal route. Therefore, it is necessary to identify anticancer drugs suitable for safe and efficacious intrathecal administration. While intrathecal administration of anticancer drugs can be effective, maintaining them at high concentrations can prove dangerous, thus adequate preventive measures must be taken. The elimination of intraventricular drugs is not uniform in all patients [1]. To safely carry out intrathecal administration of anticancer drugs, it is desirable to have a system in which local pharmacokinetics in the ventricles after administration can be evaluated [2,3,4]. The development of a method to measure the CSF concentration of intrathecally administered anticancer drugs enables chemotherapy at an appropriate dose and administration schedule for each patient.

**Figure 1:**
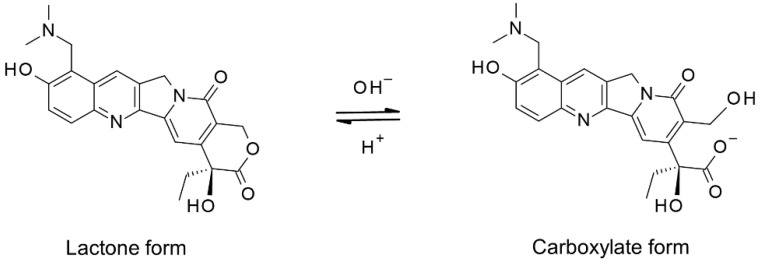
Reversible hydrolysis of Topotecan lactone ring.

Topotecan is an anticancer drug that exerts an antitumor effect by inhibiting topoisomerase I, thus preventing cell division in tumors [5]. Topotecan has been frequently used in the treatment of tumors such as rhabdomyosarcoma, medulloblastoma, and neuroblastoma in pediatrics [6,7], and its effectiveness has been gradually recognized. The structural features of topotecan are unique—lactone forms with closed lactone rings and carboxylate forms with open lactone rings exist in a reversible equilibrium under physiological conditions (Figure 1). Therefore, it is necessary to develop a drug analysis method that considers this structural characteristic of topotecan. The pharmacokinetics of topotecan concerns not only the lactone form, but also the carboxylate form. Although the lactone form is the active form of topotecan, a pH-dependent equilibrium exists between the lactone and carboxylate forms. This potential for conversion from carboxylate to lactone suggests that cytotoxic activity should be possible regardless of the proportion of topotecan in the open ring-form in the extracellular space. Therefore, total topotecan concentrations have been used to determine its pharmacokinetics [8,9,10].

Evaluation of CSF topotecan concentration after intrathecal administration is limited to phase I clinical studies [8,9,10]. Thus, personalized medicine based on monitoring CSF topotecan concentration and the necessity of daily monitoring have not been discussed to date. These issues must be addressed to further improve the efficacy and safety of intrathecal administration of anticancer drugs. Therefore, in this study, we developed a simple and highly reproducible method to measure CSF topotecan concentration, and confirmed its clinical applicability.

## 2. Materials and Methods

### 2.1. Materials

Topotecan was obtained from Sigma-Aldrich (St. Louis, MO, USA). Liquid chromatography–mass spectrometry (LC-MS)-grade acetonitrile was obtained from Merck (Darmstadt, Germany). Camptothecin, high-performance liquid chromatography (HPLC)-grade methanol, and HPLC-grade water were obtained from FUJIFILM Wako (Osaka, Japan). Dimethyl sulfoxide, acetic acid, ammonium acetate, sodium hydroxide (NaOH), and hydrochloric acid (HCl) were obtained from Nacalai Tesque (Kyoto, Japan).

### 2.2. Instrumentation

Chromatographic analysis of topotecan was performed using a Prominence Ultra-Fast Liquid Chromatography system (Shimadzu, Kyoto, Japan). Analyst software (LCsolution, Shimadzu, Japan) was used for data acquisition and analysis of chromatograms.

### 2.3. Chromatographic Separation

The separation was performed at 40 °C using an InertSustain AQ-C18 HP column (3 µm HP, 150 mm × 3 mm; GL Sciences, Tokyo, Japan). Mobile phase A was ammonium acetate buffer (75 mM, pH 4.5) and mobile phase B was acetonitrile. The chromatographic separation was achieved through gradient elution at a flow rate of 0.8 mL/min; 18.75% of mobile phase B for 4.5 min increased linearly to 43.75% in 1 min, held constant for 2.5 min, decreased to 18.75% over 1 min, and held constant for 1 min to equilibrate between injections. The detector was set at an excitation wavelength of 380 nm and an emission wavelength of 520 nm [8].

### 2.4. Stock and Working Solutions

Stock solutions of topotecan (25 mM) and the internal standard (IS) (camptothecin, 2.87 mM) were prepared in dimethyl sulfoxide and stored at −20 °C. The IS standard was diluted with water to a working solution concentration of 28.70 µM.

### 2.5. Calibration and Quality Control Samples

For drug-free CSF, samples left-over after CSF testing were used. The pooled CSF mixture from several patients was checked to ensure that it did not contain topotecan or IS. Calibrators were prepared daily by adding an accurately diluted stock solution to pooled CSF. The final topotecan concentrations in the calibration samples were 0.04, 0.08, 0.16, 0.32, 0.64, and 1.28 µM. The lower limit of quantitation (LLOQ) (0.04 µM) and three quality control (QC) samples at low (0.10 µM), intermediate (0.50 µM), and high (1.00 µM) concentrations of topotecan were prepared by adding a diluted stock solution to drug-free CSF.

### 2.6. Sample Preparation

CSF samples collected from patients receiving topotecan intrathecally were used as clinical specimens. Spiked specimens were prepared by adding an accurately diluted stock solution to drug-free CSF.

To establish the conditions for ring-form conversion of topotecan, topotecan stock solution diluted with water, topotecan-spiked CSF, and clinical CSF samples were mixed in equal amounts with water, 0.1 M HCl aquation, or 0.1 M NaOH aquation. Three hundred microliters of this mixture were then mixed with 600 µL of the mobile phase (ammonium acetate buffer (75 mM, pH 4.5)-acetonitrile (65:15, *v*/*v*)) and vigorously vortexed for 10 s. The mixture was centrifuged (14,000× *g*, 4 °C, 5 min), and 20 µL of this supernatant was injected into the chromatograph.

Based on the established conditions, the calibration samples, QC samples, or clinical samples were mixed in equal amounts with 0.1 M HCl aquation for the ring-form conversion of topotecan. Three hundred microliters of this mixture were then mixed with 600 µL of the mobile phase (ammonium acetate buffer (75 mM, pH 4.5)-acetonitrile (65:15, *v*/*v*)) and 15 µL of IS working solution and vigorously vortexed for 10 s. The mixture was then centrifuged (14,000× *g*, 4 °C, 5 min), and 20 µL of this supernatant was injected into the chromatograph.

### 2.7. Validation of the Method

In accordance with the standard guidelines for method validation [11], selectivity, LLOQ, carry-over, linearity, accuracy, precision, recovery rate, and stability were evaluated.

#### 2.7.1. Selectivity and LLOQ

Topotecan- and IS-free CSF samples pooled from several patients were used to evaluate selectivity. The lowest concentration on the calibration curve is regarded as the LLOQ. For topotecan, the interfering peak area is required to be less than 20% of the peak area of the LLOQ. For the LLOQ samples, the mean accuracy is required to be within ±20% of the nominal value, and the coefficient of variation (CV) value should not exceed 20%.

#### 2.7.2. Carry-Over and Linearity

The carry-over effects of topotecan and IS were evaluated by testing the response of a blank CSF sample immediately following the highest concentration of the calibration sample. The peak area of the blank CSF sample is required to be less than 20% of the peak area of the LLOQ sample for topotecan and 5% for the IS. The ordinary least squares method was used to fit the peak area ratio versus analyte concentration for linearity.

#### 2.7.3. Accuracy and Precision

To evaluate the accuracy and precision, five replicates of the LLOQ and QC samples at three levels were analyzed. The mean accuracy is required to be within ±15% of the nominal values for the QC samples, except for the LLOQ, which is required to be within ±20% of the nominal value. The CV values should not exceed 15% for the QC samples and 20% for the LLOQ samples.

#### 2.7.4. Recovery Rate

Three QC samples at low (0.10 µM), intermediate (0.50 µM), and high (1.00 µM) concentrations of topotecan were used to evaluate recovery rate. Topotecan was spiked at three QC concentrations in blank CSF, and spiked CSFs were treated by sample preparation described above (A). Topotecan was spiked at three QC concentrations in the final supernatant obtained by sample preparation of blank CSF (B). Three replicates of the above samples were injected into the chromatograph. The peak areas of samples A and B were obtained. The recovery rate (%) was then calculated as A/B ×100.

#### 2.7.5. Stability

Stability tests were performed by evaluating the accuracy of the QC samples at three levels under two conditions (in the CSF matrix, at 4 °C for 24 h and −20 °C for 24 h). Five replicates of QC samples at three levels were analyzed. The accuracy of the QC samples for the stability test is required to be within ±15%.

### 2.8. Clinical Monitoring of Topotecan

Clinical CSF samples were obtained from a 1-year old child receiving topotecan. The patient presented with poor absorption and stagnation of CSF due to communicating hydrocephalus after surgical resection of a brain tumor. Topotecan was administered via an intraventricular subcutaneous reservoir. The dosage was started at 0.2 mg and gradually increased to 0.3 and 0.4 mg at the discretion of the attending physician, when the adverse effects were tolerated. Informed consent was obtained from the patient’s parents to measure topotecan in the CSF. CSF samples were collected at 24, 48, and 72 h after topotecan administration at each dosage (0.2, 0.3, or 0.4 mg). The collected CSF samples were stored at −80 °C until analysis.

### 2.9. Ethics Approval

The study was conducted in accordance with the Declaration of Helsinki, and the protocol was approved by the Ethics Review Committee of the Faculty of Medicine at the University of Miyazaki, Japan (Project identification code: O-0638).

### 2.10. Statistical Analysis

R v.4.0.3. (www.r-project.org, accessed on 8 March 2021) was used for statistical analyses.

## 3. Results

### 3.1. Method Validation for HPLC

#### 3.1.1. Ring-Form Conversion of Topotecan

**Figure 2:**
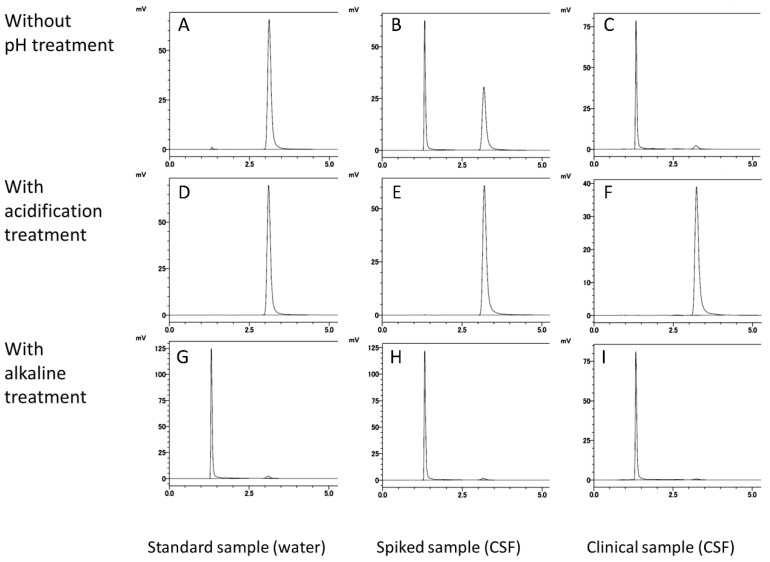
HPLC chromatograms of topotecan obtained under each pH adjustment condition. Topotecan stock solution diluted with water (**A**,**D**,**G**), topotecan-spiked CSF (**B**,**E**,**H**), and clinical CSF samples (**C**,**F**,**I**) were analyzed using the developed HPLC method. In the first step of sample preparation, samples were mixed with water (**A**–**C**), HCl aquation (**D**–**F**), or NaOH aquation (**G**–**I**).

Topotecan was converted to the lactone or carboxylate form by a simple step—adjusting the pH during sample preparation. The two forms were efficiently separated by the HPLC method we developed. The retention times of the carboxylate form and the lactone form were 1.3 min and 3.2 min, respectively (Figure 2A,D,G). Without pH adjustment, most of the topotecan was present in the carboxylate form in the clinical specimens obtained in this study (Figure 2C). In the topotecan-spiked CSF, the carboxylate and lactone forms were present at almost the abundance ratio (Figure 2B). Alkaline treatment left a small amount of lactone form (Figure 2G–I), but acidification treatment did not detect the carboxylate form (Figure 2D–F). Therefore, in this analytical system, all topotecans were converted to the lactone form by acidification treatment and quantified.

#### 3.1.2. Selectivity and LLOQ

**Figure 3:**
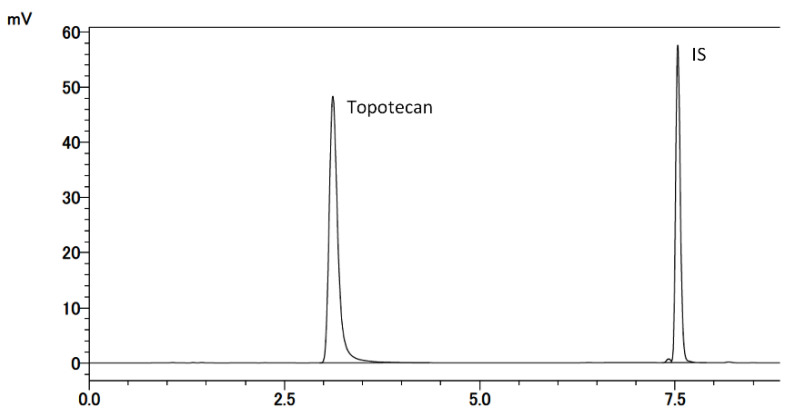
Chromatogram for the analysis of topotecan using the developed HPLC method. The retention times of topotecan (lactone form) and internal standard were 3.2 min and 7.5 min, respectively.

**Figure 4:**
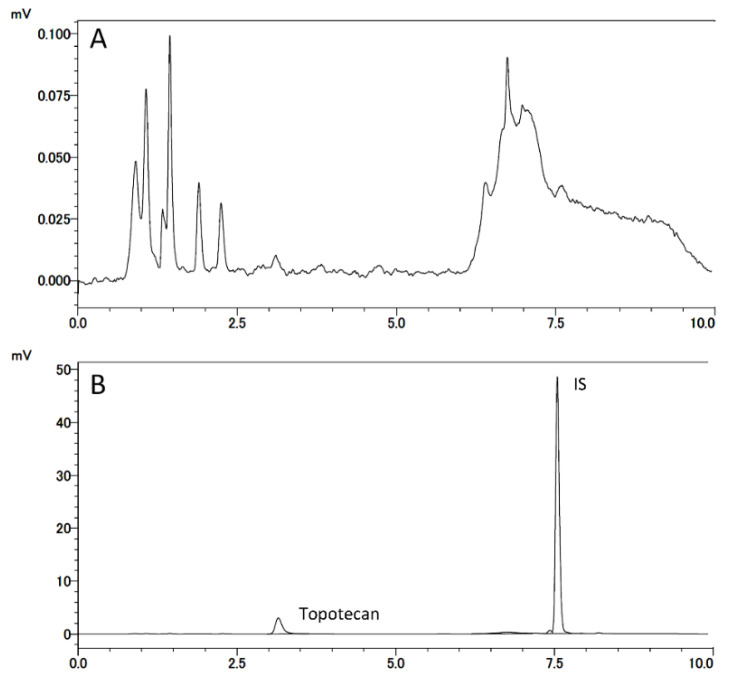
Chromatograms of drug-free CSF (**A**); lower limit of quantitation (0.04 µM) topotecan and the IS (**B**).

Typical chromatograms of the developed method are shown in Figure 3. No interfering peaks were observed in the drug-free CSF sample (Figure 4A) at retention times for topotecan and IS (Figure 4B). IS did not affect the measurement of topotecan. For the LLOQ samples, the mean relative error was 5.0% and the CV was 0.0% (Table 1).

#### 3.1.3. Carry-Over and Linearity

**Figure 5:**
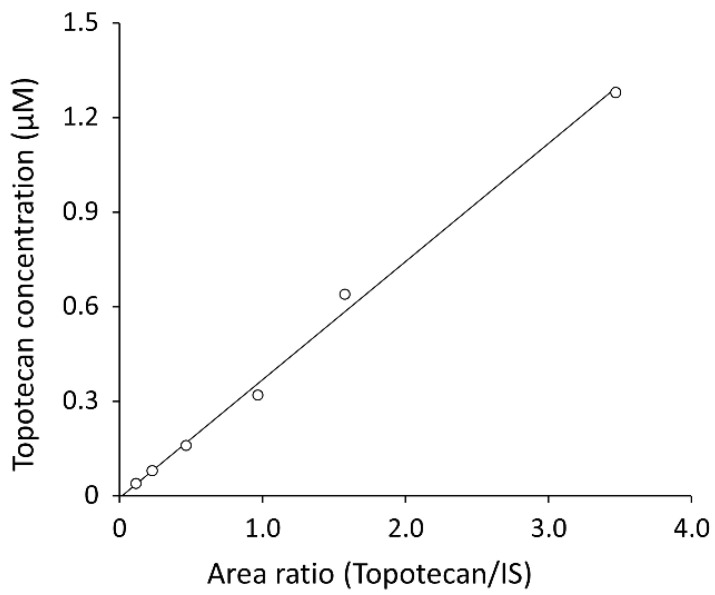
Topotecan calibration curve. A series of calibration samples were prepared using 0.04, 0.08, 0.16, 0.32, 0.64, and 1.28 µM topotecan in the CSF.

No carry-over was observed. A typical calibration curve is shown in Figure 5. Each point was a single measurement. The linear regression equation was as follows:

*y* = 0.3750*x* − 0.0057 (1)

(*x*, peak area ratio of topotecan to IS; *y*, concentration of topotecan, *r* = 0.998).

#### 3.1.4. Accuracy and Precision

The accuracy and precision values of the developed method are listed in Table 1. For the three QC sample concentrations, the mean relative error and CV were <15%. For the LLOQ samples, the mean relative error and CV were <20%.

#### 3.1.5. Recovery Rate

The recovery rate of total topotecan from human CSF at all QC concentrations was 101.2–103.6%.

#### 3.1.6. Stability

Stability tests were performed by evaluating the accuracy of QC samples at three concentrations under two conditions. The stability of topotecan in the CSF matrix at 4 °C for 24 h was evaluated based on the accuracy of low, intermediate, and high concentrations of topotecan, and the relative errors for total topotecan measurements were 0.4%, 5.5%, and 3.0%, respectively. The stability of topotecan in the CSF matrix at −20 °C for 24 h was evaluated based on the accuracy of low, intermediate, and high concentrations of topotecan, and the relative errors for total topotecan measurements were 1.6%, 0.9%, and 2.6%, respectively.

### 3.2. Clinical Monitoring of Topotecan

**Figure 6:**
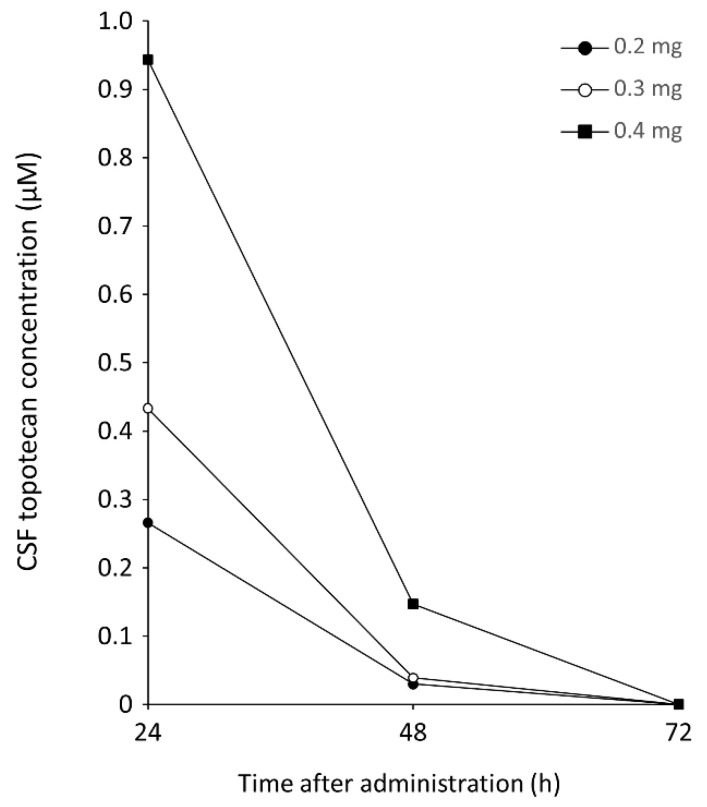
Clinical monitoring of topotecan using the developed HPLC method. The ventricular CSF concentration–time curves of total topotecan, following an intraventricular dose of 0.2, 0.3, or 0.4 mg.

In a patient with poor absorption and stagnation of CSF, topotecan was administered intraventricularly. The developed HPLC method could successfully determine the difference in concentration reflecting the dose at 24 and 48 h after administration by monitoring the CSF topotecan concentration (Figure 6). Topotecan was not detected at 72 h after administration at any dosage. In this case, the sign of adverse effect (reduced white blood cell count) was confirmed when 0.4 mg of topotecan was administered.

## 4. Discussion

We developed a simple HPLC method to measure the topotecan concentration in the CSF and confirmed that this method can be applied to clinical samples. We also developed a simple sample preparation method, including a pH adjustment method for ring-form conversion of topotecan, which can withstand the analysis of total topotecan concentration. The application of LC-MS/MS is becoming the gold standard in the analysis of drugs in biological samples [12,13]. As various drugs can be quantified by exploiting the ultra-high sensitivity of LC-MS/MS, extensive research has been used to establish analytical methods. However, for ease of operation and accessibility of devices, it is often desirable to establish an analytical method using HPLC. Therefore, the method developed in this study to measure topotecan can contribute to the realization of daily topotecan monitoring, especially because of its simplicity of operation.

Topotecan is conformationally labile in human plasma, even at cold temperatures. At −70 °C, 28% of the lactone form is converted to the carboxylate form after 2 months [14]. Therefore, to precisely analyze the concentration of the lactone and carboxylate forms in a sample, it is necessary to perform a treatment to stop hydrolysis immediately after sampling. For example, methanol treatment and preservation at −80 °C can be used. However, in daily clinical practice, it is difficult to perform such treatments each time. In contrast, the total topotecan concentration in human plasma at −30 °C does not change for at least 4.5 months [14]. Therefore, for monitoring the pharmacokinetics of topotecan in daily clinical practice, it is necessary to analyze the total topotecan concentration in the sample. In the quantification of total topotecan, the conversion efficiency between lactone and carboxylate forms in sample preparation is the key process. Lactone form predominates under acidic condition (pH < 4), whereas at pH greater than 10, the lactone ring is quantitatively opened [15,16]. At physiological pH, the equilibrium process favors the conversion to carboxylate form. By pH adjustment during pretreatment in the proposed analytical method, the conversion to the lactone form by acidification was almost complete in water and biological samples. However, in the conversion to the carboxylate form by alkaline treatment, a small amount of the lactone form remained unconverted in water and biological samples. The residual lactone form during alkaline treatment was almost constant, but it is not preferable as an analytical system to measure the total topotecan concentration due to inaccuracy. Therefore, a system was designed to quantify the total topotecan concentration after complete conversion to the lactone form by acidification.

In view of topotecan pharmacokinetics, it is necessary to understand the effects of ring-opening and -closing reactions in vivo. CPT-11, which has the same basic structure (camptothecin) as topotecan, also undergoes a pH-dependent ring-opening/closing reaction, producing a lactone form and a carboxylate form. Arimori et al. [17] reported that the open/closed state of the ring changed the pharmacokinetics of CPT-11 and its affinity for P-glycoprotein, which is responsible for the extracellular transport of CPT-11. Topotecan is also a substrate for P-glycoprotein and breast cancer-resistance proteins [18,19,20]. Among the functional components of the blood–brain barrier and the blood–CSF barrier [21], proteins of the ATP-binding cassette transporter family appear to play a significant role in transporting topotecan and are likely to affect its distribution in the brain parenchyma and CSF compartments [18,22]. Therefore, the pH of CSF may be a factor that determines the elimination of topotecan after intrathecal administration.

The correlation between CSF topotecan concentration and clinical efficacy or toxicity is still unclear. In previous studies, where topotecan was intrathecally administered and the CSF concentration was monitored, transition at a concentration of 0.1 µM or higher was reported [1,10]. Thus, the measurement method developed in this study is considered to have sufficient analytical sensitivity. In addition, the topotecan disappearance rate in the case presented in this study was extremely slow compared with that in a previous study [10]. The reason for this could be the delay in drug elimination due to poor absorption and stagnation of CSF. In a phase I trial, the maximum tolerated dose associated with chemical arachnoiditis was 0.4 mg in children [10]. However, signs of adverse effects were observed at high CSF concentrations of topotecan (0.4 mg). Therefore, as there are large individual differences in pharmacokinetics after intrathecal administration [1,23,24,25], both uniform regimen and personalized treatment development are required for the intrathecal administration of anticancer drugs. Thus, it is essential to establish and generalize the drug concentration monitoring system in the CSF. Regarding topotecan concentrations expected to have clinical effects, several cytotoxic studies have been reported in vitro [26]. However, these reports did not discuss the safety of normal tissues. In future studies, it is expected that the relationship between topotecan CSF concentration and clinical efficacy and prognosis will be analyzed by accumulating cases and performing large-scale studies.

This study had some limitations. In the proposed method, it is possible to separate lactone form and carboxylate form by chromatography, but this method does not have a sample preparation process that can reflect the abundance ratio of both forms in the living body. The LLOQ of this method needs to be further discussed in the future, because no information on the safety and efficacy of CSF topotecan concentration has been established. Ring-form conversion of topotecan by pH adjustment in this method is possible for CSF samples, but it has not been verified whether it is an analytical method applicable to other biological samples such as serum and urine. Components such as proteins and lipids can affect conversion efficiency.

## 5. Conclusions

We developed a method to measure the total topotecan concentration in the CSF; this method can sufficiently detect the delay in topotecan elimination after intrathecal injection. The realization of daily topotecan monitoring enables timely adjustment of the topotecan dosage and injection interval for intrathecal administration of topotecan. The findings of this study are valuable for the development of personalized treatments for the intrathecal administration of anticancer drugs.

## Figures and Tables

**Table 1:** Sensitivity, accuracy, and precision of the HPLC method in the measurement of topotecan concentration in human cerebrospinal fluid.

Concentration (µM)			
Spiked	Measured (Mean ± SD)	CV (%)	Relative Error (%)
0.04	0.038 ± 0.000	0.00	5.0
0.10	0.086 ± 0.001	1.51	13.8
0.50	0.469 ± 0.006	1.28	6.2
1.00	0.921 ± 0.005	0.52	7.9

*n* = 5; SD: standard deviation, CV: coefficient of variation.

## Data Availability

The data presented in this study are available on request from the corresponding author.
